# Patients’ perceptions of climate-sensitive health counselling in primary care: Qualitative results from Germany

**DOI:** 10.1080/13814788.2023.2284261

**Published:** 2023-11-27

**Authors:** Silvan Griesel, Patricia Nayna Schwerdtle, Claudia Quitmann, Ina Danquah, Alina Herrmann

**Affiliations:** a Heidelberg Institute of Global Health (HIGH), University Hospital Heidelberg and Medical Faculty of Heidelberg University, Heidelberg, Germany; b Interdisciplinary Center for Scientific Computing, Heidelberg University, Heidelberg, Germany; c School of Nursing and Midwifery, Faculty of Medicine, Nursing and Health Sciences, Monash University, Melbourne, Australia; d Institute of General Medicine, University Hospital Cologne and Medical Faculty of Cologne University, Cologne, Germany

**Keywords:** Climate change, patient education, climate-sensitive health counselling, health communication, patient-centeredness, primary care, general practice

## Abstract

**Background:**

Climate change is the greatest threat to global health in the twenty first century, yet combating it entails substantial health co-benefits. Physicians and other health professionals have not yet fully embraced their responsibilities in the climate crisis, especially about their communication with patients. While medical associations are calling on physicians to integrate climate change into health counselling, there is little empirical evidence about corresponding perceptions of patients.

**Objectives:**

This study aimed to explore primary care patients’ perceptions of climate-sensitive health counselling.

**Methods:**

From July to December 2021, 27 qualitative interviews with patients were conducted and analysed using thematic analysis. A purposive sampling technique was applied to identify patients who had already experienced climate-sensitive health counselling in Germany.

**Results:**

Patients’ perceptions of climate-sensitive health counselling were characterised by a high level of acceptance, which was enhanced by stressing the link between climate change and health, being credible concerning physician’s own climate-friendly lifestyle, building upon good therapeutic relationships, creating a sense of solidarity, and working in a patient centred manner. Challenges and risks for acceptance were patients’ disinterest or surprise, time constraints, feared politicisation of consultations, and evoking feelings of guilt and shame.

**Conclusion:**

These findings suggest that primary care patients can accept climate-sensitive health counselling, if it follows certain principles of communication, including patient-centredness. Our findings can be useful for developing communication guidelines, respective policies as well as well-designed intervention studies, which are needed to test the health and environmental effects of climate-sensitive health counselling.

## KEY MESSAGES

Climate-sensitive health counselling was accepted in a qualitative patient sample in Germany.

Patient-centred communication and a link to individual health contributed to acceptance while time-constraints, politisation and feelings of guilt were potential challenges.

Further research is needed to investigating patients’ acceptance and effects of climate-sensitive health counselling in larger samples.

## Introduction

‘I will respect and honour the trust placed in me and leverage this trust to promote knowledge, values, and behaviours that support the health of humans and the planet’. In 2020, this sentence was proposed as an update of the Hippocratic oath in the Anthropocene, the age in which humans significantly impact Earth’s systems, overcrossing planetary boundaries and endangering planetary health [1]. In the spirit of this pledge, numerous health and medical societies [2, 3] explicitly call on health professionals to integrate climate change and health into health communication with patients. Indeed, climate change is a significant threat to human health. This includes direct impacts such as extreme weather and heat waves, and indirect impacts such as changing patterns of infectious diseases and allergies as well as deteriorating air pollution, food quality, and psychosocial stress [4]. Along with that, tackling climate change can improve global health *via* the health co-benefits of mitigation by promoting plant-based diets, increasing active travel, and reducing air pollution [5].

A recent scoping review has defined the term ‘climate-sensitive health counselling’ as integrating climate change and health topics into communication between patients and health professionals in routine healthcare activities [6]. It evaluates the evidence around CSHC and elaborates on content areas, communication strategies as well as barriers and facilitators. Examples of contents that can be integrated into CSHC include the impacts of climate change on (patients’) health and respective adaptation measures, healthy and climate-friendly lifestyles (e.g. diet, mobility) and climate action and policies. Some suitable communication strategies identified were patient-centred communication, motivational interviewing and emphasising co-benefits for climate and health.

So far, studies show that health professionals feel responsible for conducting CSHC but do not implement it [7–11]. One main barrier is high uncertainty about patients’ perceptions of CSHC: In several studies, physicians and other health professionals express the fear that patients might perceive climate change to be too politicised for the clinical setting [7–9, 12]. Moreover, they worry it could compromise the therapeutic relationship [8, 12] or expect low interest among patients [7]. Lastly, they feel unknowledgeable about how to approach patients regarding climate change [9].

This uncertainty is also due to a lack of literature on patients’ perspectives on CSHC [8, 10, 13]. One study in the USA showed that most parents in paediatric practices were in favour of being educated about the impact of climate change on their children’s health but did not want to hear about climate policy engagement [11]. In another study, a standardised message about the effects of climate change on child health was generally well-received [14]. Yet, more research is needed on how adult patients accessing primary care respond to integrating climate change and health issues into health counselling. Due to their unique access and proximity to patients and communities, general practitioners are well-placed to provide CSHC [7]. Therefore, this study aims to explore patients’ perceptions of CSHC conducted by primary care physicians in Germany.

## Methods

### Study design

We chose a qualitative study design applying semi-structured interviews with patients who experienced CSHC. The qualitative design allows to explore CSHC openly, given that this is an emerging social phenomenon in primary care. Ethical approval for this study has been obtained from Heidelberg University’s ethics review board (S-917/2020).

### Sampling and eligibility criteria

We used purposive sampling because we needed to identify patients who already had experienced CSHC, as defined in the introduction, with a primary care physician in Germany, which was the main eligibility criterion. Furthermore, patients had to be over 18 years of age and provide written informed consent to participate in the study. Maximum variation sampling was applied to cover diverging perspectives of patients and create an ‘information-rich’ sample [15]. Variation was sought for patients’ characteristics that we assumed to influence patients’ perceptions of CSHC, such as age, sex, socio-demographic status, climate change-related attitudes. To assess those characteristics a short quantitative survey covering patients’ socio-demographic characteristics and a standardised tool to classify climate change-related attitudes (Global Warming Six Americas, GWSA) [16] (Supplementary Material 1) was administered to participants.

### Recruitment

Patients were retrospectively recruited between July and December 2021 in six German primary care practices by physicians who were experienced in CSHC. To identify such physicians, we sent open invitations to participate in the study through the section Climate Change and Health of the German Society for General and Family Medicine (DEGAM) and the network of the non-governmental organisation German Alliance of Climate Change and Health (KLUG e.V.). Six physicians with various profiles for age, sex, work experience, and geographical location were chosen for recruitment (Supplementary Material 2). These physicians identified eligible patients who had received CSHC with the physician and supported the recruitment process as detailed in Supplementary Material 3. All patients who had contact with the study team received oral and written study information and were asked to sign an informed consent form if they wished to participate. We regularly reviewed the short survey on patients’ characteristics to check, reaching a sample with maximum variation.

### Data collection

Interviews were conducted using a semi-structured interview guide (Supplementary Material 4). The guide was developed using the ‘collect, review, sort, subsume’ – method, according to Helfferich [17, p. 182 ff]. It was pilot-tested with a convenience sample of two testers aged 29 and 57 years. In addition, the interview guide was slightly adapted during the first 5 interviews to follow new lines of inquiry where necessary. One researcher (SG) carried out all interviews *via* telephone and video call. Interviews were transcribed during the data collection and notes were taken. Data saturation was determined bilaterally by SG and AH around the 25th interview as the degree of repetition across the data set regarding essential aspects of patients’ perceptions of CSHC was considered sufficient. Two more interviews were conducted to ensure that no further aspects emerged.

### Data management and analysis

The questionnaire on patients’ characteristics was analysed using descriptive statistics with SPSS (version 26 in Windows 10) and the GWSA data analysis was supported by the respective coding manual [18].

All interviews were audio-recorded and transcribed verbatim using the transcription rules provided by Dresing et al. aided by f4x automatic transcription software [19]. Using an inductive approach, we conducted a thematic analysis according to Braun and Clarke [20]. We favoured this approach because its applicability in life sciences has been shown in several studies, including evaluation of health interventions in primary care settings [21].

We followed the six iterative steps of thematic analysis: (1) familiarisation with the data (2) generating initial codes relevant to the research question, (3) searching for themes by grouping the codes identified, (4) reviewing themes to coded data, (5) defining and naming themes, and (6) producing the report.

A junior researcher (SG) conducted the analysis as a primary coder, mainly using inductive and semantic coding aided by NVivo 12 Pro software. A senior researcher (AH) listened to five interview transcripts and met with SG weekly during the analysis. CQ helped to validate the final coding scheme. Two other senior researchers (PNS, ID) were part of monthly team meetings to review, define and name themes. Following an iterative data analysis approach, we specified our broad research objective of exploring patients’ perceptions of CSHC by defining two sub-objectives ('investigating factors contributing to acceptance;’ ‘identify risks and challenges for acceptance’) to reflect better the theme of acceptance discovered in the analysis. We reviewed acceptability frameworks to evaluate their usefulness for data analysing [22]. However, we decided to take an inductive approach to the data analysis because the interviews revealed that CSHC was not delivered as a well-defined intervention that patients could rate along the lines of existing acceptability frameworks.

### Research team and reflexivity

The first author SG, a male physician without qualitative research experience, attended several methods courses to gain the necessary expertise. The other co-authors have diverse backgrounds such as medicine, nutrition, epidemiology, nursing and health promotion and were experienced in qualitative research. There was no prior relationship between patients and research team members. Patients knew SG was a physician working on his doctoral thesis.

SG, CQ and AH are part of the non-governmental organisation (NGO) KLUG e.V., and AH is the DEGAM Section on Climate Change and Health spokesperson. Through these communities, they were made aware that physicians were practising CSHC and got interested in exploring this phenomenon scientifically. Some recruiting physicians were professionally known to the authors through those networks. Thus, the research was related to the authors’ NGO membership and SG, CQ and AH share the values of the climate change and health community, mainly promoting climate change adaptation and mitigation in the health sector. At the same time, the respective co-authors reflected critically about their roles as researchers and NGO members and paid particular attention to exploring patients’ perceptions of CSHC in an open and non-judgmental way throughout the research process.

## Results

### Patient characteristics

After the recruitment process (Supplementary Material 3), the final sample consisted of 27 patients, who covered a wide range of patient characteristics as detailed in Table 1.

**Table 1.** Characteristics of patients and interviews.

Characteristics	median (minimum – maximum)/ percentage (*n*)
**Demographic characteristics**	
*n*	27
Age (years)	62 (18 – 89)
Male sex	48% (13)
**Socioeconomic characteristics**	
Education	
* *Major/secondary school diploma AND no completed vocational training	7% (2)
* *High school diploma OR completed vocational training	56% (15)
* *University degree at bachelor level, completed master school or administrative college or similar	22% (6)
* *University degree at master level or similar, doctorate, post-doctorate	11% (3)
Climate change-related attitudes according to the ‘Global Warming Six Americas’	
Alarmed	33% (9)
Concerned	48% (13)
Cautious	7% (2)
Disengaged	7% (2)
Doubtful	4% (1)
Dismissive	0% (0)
**Interview and study specific characteristics**	
Interview duration (minutes)	66 (28 – 112)
Interview mode	
* via* telephone	63% (17)
* via* videocall (Webex by Cisco)	37% (10)
Days between CSHC and the interview	19 (5–264)
Patients per recruiting physician	5 (2–6)

### Findings of thematic analysis

Overall, patients’ perceptions of CSHC in this sample were characterised by high acceptance. Acceptance was identified as the central theme because it captures patients’ latent agreement and approval of CSHC across the whole dataset. Yet, patients did mention some challenges and risks for acceptance of CHSC. The identified risks were not always based on patients’ experience related to CSHC but also on hypothetical and scenarios with which patients were prompted in the interview (see Supplementary Material 4). The results section presents the main themes of ‘acceptance’ and ‘challenges and risks to acceptance’. If the reader wants to understand the actual contents of CSHC first, topics addressed during CSHC are summarised in Supplementary Material 5.

## Acceptance

Acceptance was often shaped by how physicians delivered CSHC and patients’ prior attitudes and perceptions. Table 2 summarises the sub-themes and quotes identified as factors contributing to acceptance of CHSC.

**Table 2.** Sub-themes and exemplary quotes for contributors to acceptance.

Sub-themes	Quote
Link to health (1)	‘She said, given my blood count it would make sense to do this and that. And then her formulation was […]: In this way you not only serve your health, but also […] the planet’s health […]. She practically introduced the political side of these questions through the question of the patient’s health. ‘(P11, m, 65)
Patient-centredness (2)	‘The physician approached this slowly to see whether this person [the patient] was the right one […] whether it was suitable or had a VALUE to talk about it. […]. By the fact that I took up the conversation, […] she noticed: Okay, I am not dismissive […] and that it is not uninteresting [for me]’. (P22, m, 32)
Physician’s credibility (3)	‘I MEAN to have heard from the conversation that she also eats differently, so more environmentally conscious […] And as I said, she now goes on home visits by bike, I have seen that […] And so I thought: That was the right contact person […] [She] is also a physician who LIVES what she says, […] so she is someone who can be easily followed because she represents this authentically for me’. (P4, f, 62)
Trustful physician-patient relationship (4)	‘The [physician-patient relationship] has been strengthened, it has almost turned into a friendship-like relationship. Because you can feel some kind of wavelength. […] You just like to surround yourself with people like that. […] That you feed each other a little bit and complement each other. […] I would say it’s a story of equals’. (P5, f, 58)
Sense of responsibility (5)	‘It always starts on a small scale with ourselves. And that […] could still be seen as apolitical. That is just reasonable, […] because we all, including me, have to take care, that our world is still worth living’. (P13, f, 61)
Sense of solidarity (6)	‘When you sense and feel […] that other people are also thinking and working in this direction and trying to move something. That just does a lot of good. You then feel like a PART of the whole. […] Wow, […] other people are fighting, TOO’. (P15, f, 54)
Holistic and caring perspective (7)	‘It is very pleasant when someone simply looks further and thinks ahead. And just this comprehensiveness. […] Wow, this is someone who cares about me […] who WORRIES about me. Not just about my arm, but simply about ME entirely’. (P15, f, 54)

### Link to health (1)

For many patients in this study, the link between climate change and health legitimised discussing climate change during health counselling. Patients recognised a responsibility of physicians to educate them about the health impacts of climate change and the health co-benefits of climate-friendly lifestyles, thereby strengthening patients’ health literacy and autonomy. Although some conversations went beyond health-related aspects, for most patients, the conversation had to be focussed on health issues or even the primary health concern that had led them to consult the physician.

### Patient-centredness (2)

Most patients felt that CSHC had to be adapted to the patient’s interests, values, and current biopsychosocial context through individualised and empathetic communication. Patients appreciated not being forced into the conversation, some indicated that physicians tried to determine whether they were receptive to the topic. For instance, two patients appreciated a conversation about plant-based diets because it strengthened their motivation to change their dietary behaviour.

### Physician’s credibility (3)

The credibility of physicians regarding climate change-related issues was important. Few patients explicitly mentioned their high trust in physicians due to their societal role, and high level of education. More often, however, patients appreciated if their physician acted authentically, i.e. seemed enthusiastic and convinced but also emotionally concerned, and had an authentic, climate-friendly lifestyle that served as a role model for patients.

### Trustful physician-patient relationship (4)

A good physician-patient relationship was sometimes mentioned as a prerequisite for CSHC. However, several patients expressed a sense of agreement with the physician on the issue of climate change, of being on *the same wave length,* which seemed to have strengthened their relationship with the physician. Moreover, a personal and trusting exchange *at eye level* and the experience of shared learning, contrast to a paternalistic physician-patient relationship, was described as central to the acceptance of CSHC. While no patient reported that CSHC damaged the physician-patient relationship, some argued the relationship had even improved.

### Sense of responsibility (5)

Many patients in this study saw climate change as an urgent issue and emphasised that each individual was responsible for contributing to climate action. Therefore, these patients considered it legitimate for the physician to appeal to this responsibility, to create awareness and even suggest behaviour change.

### Sense of solidarity (6)

Some patients expressed gratitude for the opportunity to exchange ideas on climate change. They felt accompanied by their physicians in their thoughts, feelings, and worries about climate change as well as in their efforts in climate action. They found it valuable and motivating to see these mirrored and confirmed by their physician.

### Holistic and caring perspective (7)

Some patients felt that physicians expressed a caring attitude and holistic perspective on health by conducting CSHC, which led to patients experiencing *comprehensive* counselling that incorporated the *whole person*. The impression that the physicians took their time with them was necessary in this context.

### Challenges and risks to acceptance

Despite the general acceptance of the experienced CSHC in this sample, some disconfirming cases, summarised in the theme ‘challenges and risks to acceptance’. Table 3 summarises the subthemes and quotes identified as ‘challenges and risks’.

**Table 3.** Sub-themes and exemplary quotes for challenges and risk to acceptance.

Sub-theme	Quote
Time constraints (8)	‘Everyone knows that the physician only has a certain amount of TIME for the patient. […] The physician must first deliver on the reason you came to him. And after that he can also have such a conversation, […] if he has the TIME for it [and] the patient does not suffer from it’. (P22, m, 32)
Getting too political (9)	‘She didn’t take it in a political direction. So it wasn’t about WHAT concrete measures have to be taken and which party (laughs) is better than another. It was only about pure facts; if they are scientific facts, then I don’t see this as a political issue at all’. (P29, m, 26)
Guilt, shame, questioning individual responsibility (10)	‘And I find […] that this is being laid upon our shoulders. And we have to start with ourselves. But again, I also EXPECT the state to protect me and deal with the issue. […]. And clearly, the role of GP I think is super important […] Who else does the patient BELIEVE if not the GP?’ (P20, w, 52)
Disinterest (11)	‘I remember thinking again: ‘My God, now it’s climate change again, and possibly only man-made’, [and] that I'm sick of it’. (P24, m, 78)
Surprise (12)	‘And that left me a bit STUNNED, […] this commitment. [The GP] advocated for a [climate] demonstration and so on. […] I thought it was great, I thought it was GOOD. I was just surprised because you are not prepared for it, that it comes suddenly’. (P6, w, 62)

#### Time constraints (8)

Due to existing time constraints, many patients questioned a larger-scale inclusion of climate change and health topics in the consultation. Thus, some patients feared that talking about climate change would compromise adequately addressing the patient’s health concerns, although no patient experienced that.

#### Getting too political (9)

Most patients in this study did not perceive CSHC as politically inappropriate. Talking to patients about climate change and health was not perceived as political. Yet, the confrontation with hypothetical scenarios of physicians talking about aspects that touched upon political aspects of climate change in the interview guide often triggered unease. However, patients who discussed more political topics during CSHC (i.e. climate mitigation policies and participating in climate demonstrations) usually perceived it as appropriate. Patients clearly did not wish to be directly influenced by their political attitudes; for instance, they did not want to receive voting recommendations.

#### Guilt, shame, questioning individual responsibility (10)

A few patients questioned individual responsibility regarding climate action and put it into perspective with little governmental or corporate action, and barriers for individual engagement (e.g. poor local public transport, time constraints, struggling with change of habit). Therefore, they found it critical to transfer the responsibility for lifestyle change upon the individual, which could happen during CSHC. For some, this sense of personal responsibility for climate action made them feel guilty and ashamed.

#### Disinterest (11)

Few patients expressed disinterest or rejection of CSHC in this sample. For instance, two participants denied anthropogenic climate change. However, they did not explicitly criticise their physician for CSHC but did not attach much importance to their physician’s statement about climate change.

#### Surprise (12)

Some patients expressed surprise about CSHC because they perceived the topic of climate change as atypical and rare during a consultation. While the surprise was not necessarily experienced as unpleasant, it did represent some level of irritation.

## Discussion

### Main findings

Overall, patients in this study broadly accepted CSHC. This acceptance was associated with linking climate change to personal health issues and using patient-centred communication in a trusting physician-patient relationship. We also identified challenges and risks of CSHC, including perceived time constraints, getting too political during CSHC, and transferring too much responsibility for climate change mitigation upon the individual.

### Factors contributing to or challenging acceptance

Maintaining a solid reference for (individual) health was an essential factor contributing to acceptance of CSHC. This relates well to other research, which shows that focusing on individual health concerns increases the acceptability of lifestyle interventions overall [21]. Furthermore, other research on CSHC by physicians underlines the importance of this approach [8, 23]. Alame et al. even argue that the responsibility of physicians within climate change communication needs to be limited to health concerns [24].

Patients implied that CSHC was more acceptable if delivered in a patient-centred manner. This is also reflected in other qualitative studies exploring health professionals’ views on CSHC [8, 12] and existing CSHC guidelines [6]. While the inductive sub-theme of patient-centeredness in CSHC for us meant focussing the conversation on patients’ attitudes, interests and current situation, there are broader concepts for patient-centred communication (PCC) in the literature. According to Epstein, PCC (1) elicits patients’ perspectives, (2) understands the patients within their psychosocial context, (3) reaches a shared understanding of the problem and its treatment concordant with patient’s values, and (4) helps patients to share power and responsibility to the degree that they wish [25]. This concept also touches upon several other sub-themes identified in this study.

For instance, the PCC concept emphasises to act according to patients’ values and enabling them to share responsibility ‘to the degree that they wish’. This could be applied to patients’ values and responsibility for climate change. In the context of the sub-themes ‘sense of responsibility’, ‘disinterest’, ‘getting too political’ and ‘guilt, shame and questioning individual responsibility’, the recommendations for PCC could mean that physicians should elicit patients’ values and sense of responsibility in climate action to find out how deep they can enter into the topic of climate change and health. If the physician senses the patient sees climate change as an urgent issue or perceives personal responsibility for climate change, this would be a cue to explicitly mention the climate benefits of healthy lifestyles, such as plant-based diets and active mobility. In a Special Eurobarometer on climate change from 2021 56% of Germans and 41% of Europeans thought that within the European Union (EU) they are responsible for tackling climate change and 78% of Europeans thought climate change to be a severe problem (79% of Germans) [26]. Yet, caution should be taken in shifting blame for climate change on individual patients, given the scale and structural nature of the climate crisis. One study warns against making patients feel guilty about climate change [27], which aligns with our finding that creating guilt and shame could challenge the acceptance of CSHC.

Concerning the risk of CSHC getting too political, the principle of acting concordant with patients’ values in PCC also proves helpful [25]. For example, in a survey conducted in Pittsburgh, USA, Ragavan et al. found that 64% of parents strongly disagreed that their paediatrician should talk to them about political engagement related to climate change (‘talking to decision-makers about reducing global warming’) [11]. Yet in our study, CSHC involving discussion about political engagement was mostly found to be appropriate. One possible reason for this could be that physicians were practising CSHC patient-centred and only discussed political issues with patients, whose attitudes and values opened the door for such a conversation.

A trustful physician-patient relationship is central to elicit patients’ perspectives, appreciate their psychosocial context and reach a shared understanding of a problem. Contrary to the apprehension of some physicians [8, 12], our findings add weight to the notion that CSHC does not endanger an excellent therapeutic relationship but can potentially strengthen it [24], particularly if patients and physicians share similar attitudes towards climate change. Previous literature has found that personal connection and continuity of care, as given in general practice, can serve as a prerequisite to talk about more sensitive topics and behaviour change [21]. However, patients’ experience of CSHC in this study partly went beyond the concept of PCC because patients felt that physicians did not only search for a shared understanding of the problem but were even willing to learn from patients. Guggenheim supports this idea by understanding patients as potentially ‘motivated and knowledgeable partners in efforts for sustainable living’ [13]. A stance of shared learning between partners in dialog is also recommended in general climate communication guidelines and should also be integrated into CSHC [^28^]. This is also reflected in the sub-theme ‘sense of solidarity’ where patients appreciate their physicians sharing worries and thoughts about climate change.

Similarly, patients appreciated ‘physicians’ credibility’ and seeing climate-friendly lifestyles or engagement for climate action in their physicians contributed to acceptance of CSHC. Being authentic about one’s own lifestyle is known to be the basis for credibility in climate change communication and lifestyle guidance in health counselling [21, 29].

Finally, patients in this study identified a lack of time as an important challenge of CSHC, which is common found barrier for CSHC in surveys with health professionals [6–8]. Patients feared that their health concerns could receive too little attention compared to climate change. However, no patient reported that they experienced how time constraints compromised the treatment of their health issue. This underlines other conceptual claims, saying that CSHC can entail short messages and be integrated into standard counselling procedures [6,29].

### Strengths and limitations

To our knowledge, this is the first study exploring primary care patients’ perceptions of CSHC in Germany. A strength of this study is that we were able to interview patients who had experienced CSHC in a real-life setting provided by physicians experienced in CSHC. This provides empirical evidence to contribute to the conceptualisation of CSHC from a patient’s perspective. The purposeful selection of physicians *via* climate change and health networks for the recruitment of patients might have led to the choice of a specific study population of patients. Yet, qualitative research is not meant to give generalisable results, such as how many patients find CSHC acceptable. Rather, qualitative research wants to create information-rich samples, which allow the purpose of the study [15], which in this case was to explore patients’ perceptions of CSHC. The study participants’ heterogeneous attitudes towards climate change and CSHC show that we fulfilled this aim of maximum variation sampling. While we have captured various patients’ perceptions, we identified a standard set of factors contributing to or challenging acceptance of CSHC. We could show in the discussion that many of these factors resonated well with findings in the international literature. This suggests some transferability to other European primary care contexts and possibly elsewhere.

### Implications for research and practice

The way in which patients communication about political or polarising issues depends on the communication skills of practitioners [31]. To make the results of this study actionable for general practitioners, the research team has summarised practical recommendations for CSHC in Box 1, drawing on results from this study and findings from the existing literature as elaborated in the discussion.

Patients’ acceptance of CSHC following these recommendations needs to be further investigated with qualitative studies in other contexts of primary care in Europe and by using quantitative studies with larger sample sizes. Moreover, intervention studies should measure the actual effects of CSHC on health and climate change-related outcomes, like climate change and health knowledge, attitudes towards climate action, or healthy and climate-friendly behaviour.

Box 1 Recommendations for ‘Climate-sensitive Health Counselling’ drawn from the presented results and their discussion with literature. The most relevant sub-themes resulting into the recommendation are mentioned in bracket as numbered in Tables 2 and 3.

**Table:** 

Make a link to health, ideally the individual health concern of the patient. (Sub-theme 1, 2, 7, 11) Communicate in a patient-centred manner, which means adapting CSHC to patients’ attitudes, values, and their current bio-psycho-social context. (Sub-theme 2, 5, 8, 9, 10, 11) Choose a flexible timeframe: Start with short messages and enter into a deeper conversation if patients’ (health) interests allow. (Sub-theme 2, 8, 11) Act authentically through engagement in climate action in your own life. (Sub-theme 3) Talk at eye level, share your own thoughts and feelings around climate change and show a willingness to learn from patients. (Sub-theme 4, 6) Deliver facts on climate change and health in a politically neutral manner and avoid making the conversation partisan or overly political. (Sub-theme 9) Acknowledge shared responsibility in climate action and avoid triggering feelings of guilt and shame in patients. (Sub-theme 5, 6, 10)

## Conclusion

Patients’ acceptance of CSHC provided in real-life settings in primary care in Germany suggests that the call of health organisations to integrate climate change and health issues into health counselling is feasible. Yet, factors contributing to or challenging patients’ acceptance of CSHC must be considered by health professionals to maximise acceptance. The results of this study can help to further conceptualise and implement CSHC in research and practice. Finally, intervention studies are needed to evaluate the actual health and environmental effects of CSHC.

## Supplementary Material

Supplemental Material

Supplemental Material

Supplemental Material

Supplemental Material

Supplemental Material
