# Supplementary material for: Patients’ perceptions of climate-sensitive health counselling in primary care: Qualitative results from Germany
Source: Eur J Gen Pract. 2023 Nov 27;29(1):2284261. doi: 10.1080/13814788.2023.2284261 (PMC10773651; doi:10.1080/13814788.2023.2284261)
Supplement: Supplemental Material [file IGEN_A_2284261_SM2825.docx]

### **Supplementary Material 5. Topics addressed during CSHC**

Participants reported a diverse set of topics related to climate change and health addressed during health counselling with their physician, as depicted in Table 1.

Table 1: Subthemes and exemplary quotes for topics during CSHC

| **Subtheme** | **Quote** |
| --- | --- |
| Climate change in general | *‘Where all this should lead to with the warming up [...], also with all the waste […], that resources are thrown away and so on. That’s what we talked about. And then we always got a bit further, right? [...] ‚Wow,‘ I say, ‚the doctor is also thinking SERIOUSLY about climate change and where we should go from here.’* (P2, m, 63) |
|  |  |
|  |  |
|  |  |
| Lifestyle change | ‘*That's how she [the physician] put it: It's better to go by bus or by bike […] to the city to do the shopping etc. And there one would do MUCH, much good for climate change’* (P10, m, 67) |
|  |  |
| Health impacts and adaptation measures | ‘*Two days later, this intense heat returned for two or three days. And then she [the physician] just told me that if something is wrong, I pause the medication [...] for the blood pressure. And THAT'S when we came [to climate change]. [...] Drink a lot, keep calm, don't do anything, stay inside because that impacts the body.*’ (P7, f, 62) |
|  |  |
|  |  |
|  |  |
|  |  |
| Personal engagement (non-political) | ‘*And [then we] also came to the topic of climate change in the context of care for the elderly. [...] She [the physician] asked how things are going in the elderly home [where the participants works as a nurse] and (sighs) IF anything is being done there and what I thought one COULD do.’* (P15, f, 54) |
|  |  |
|  |  |
|  |  |
| Climate action and policies | ‘*We had [...] talked about it, about this Friday [for] Future because I had said that my son is also interested and involved in something like that [...] And then she started talking about it and said: ‘I'm doing this’ […] and ‘What do you think about that? [...] Maybe you* *can get involved.’* (P12, f, 42). |
|  |  |
|  |  |
|  |  |

The most common topics were climate-friendly lifestyles, climate change’s health impacts and respective adaptation measures. Concerning lifestyle recommendations (i.e. meat-reduced diets, active mobility by bike), physicians often seemed to start talking about the health effects for their patients and then they mentioned the co-benefits for the environment or the planet. The topic most commonly described about health impacts of climate change was heat, followed by air pollution, infectious diseases, and mental health. How to adapt to these impacts to protect patients’ health were often addressed. While lifestyle and health impact contents had a close relation to health, patients also reported that the conversations expanded to more general issues around climate change, such as public exchange about extreme wheather events, societal developments contributing to climate change, or concerns around the seriousness of the issue.

In a few cases, patients described explicitly discussing possibilities for personal engagement beyond changes in lifestyle and consumer behaviour, for example in the work environment. In rare cases, conversations about climate action and other political aspects were described. Patients had experienced that their physicians shared their personal engagement in climate action and invited patients to participate.
