# Supplementary material for: Patients’ perceptions of climate-sensitive health counselling in primary care: Qualitative results from Germany
Source: Eur J Gen Pract. 2023 Nov 27;29(1):2284261. doi: 10.1080/13814788.2023.2284261 (PMC10773651; doi:10.1080/13814788.2023.2284261)
Supplement: Supplemental Material [file IGEN_A_2284261_SM2808.docx]

**Supplementary Material 4. Interview Guide**

English Translation (Original in German language)

Interview guide for patient study on climate-sensitive health counselling

*Participant and encounter data:*

Interview ID:

Date of the interview:

Interviewer:

*Postscript:*

Interview atmosphere & relationship:

*Course of interview (dynamics):*

*Peculiarities (e.g. striking themes, disturbances, gestures):*

*Other:*

| **Introduction** |
| --- |
| 1. **Welcome and introduction** |
| 1. **Repeating information for patients**    - Topic: Climate change and health in GP counselling    - Study aim:      1. Investigating patients' attitudes, thoughts and perceptions concerning talking about climate change during the patient encounter.      2. Developing guidelines for the GP-patient encounter on the issue of climate change |
| 1. **Repeating information on ethical aspects**    - Tape recording or audio recording via online tool    - Data protection    - Voluntariness and possibility of revoking the declaration of consent    - The results will only be published anonymously, so that no statements can be traced back to you personally. We will not disclose the contents of this encounter to anyone, not even your doctor.    - Do you have any questions? |
| 1. **Notes on the encounter procedure:**     - Duration approx. 1 hour    - Guide contains topics to be covered in the encounter but also leaves room for unforeseen content.    - Mainly open questions, follow-up questions possible at any time!    - The interview is not a knowledge test. There is no ‘right’ and ‘wrong’ but it is about examining YOUR perceptions and thoughts. We want to learn from YOU    - Questions should be understood as a narrative stimulus    - Interview not the same as normal conversation: Interviewer's reaction might seem somewhat reserved but this is not an expression of disinterest    - Breaks for reflection - even longer ones - perfectly fine    - Memory gaps are entirely normal. Let's see what else we can find together!    - Do you have any questions?   x Check consent form x  x Encounterer: Start audio recording x |

| **Socio-economic data (see Survey for other items)** |
| --- |
| 1. **State, city:** 2. **Living environment (urban vs. rural):** 3. **Current/former occupation, brief description of educational background (school-leaving qualifications, vocational training, intended employement):** 4. **Do you have children/ grandchildren?:** 5. **Date of birth:** 6. **When was the encounter of interest with the doctor?:** 7. **How long have you been a patient of your doctor?:** |

| **1. General contents of the encounter with your doctor** | |
| --- | --- |
| **We asked you for the interview because your doctor told us you were eligible for this study. Perhaps you could first describe from your perspective what you talked about with your doctor during the respective encounter with him/her?** | |
| Content aspects | Possible further enquiries |
| - Basic contents of the encounter - Experienced focal points of the encounter - Active reminder of the encounter | - What were the basic contents of the encounter? - What can you still remember? - What was the course of the encounter? - What else do you remember? - In your memory, what was the focus of the encounter? |

| **2.1 General experience of the encounter** | |
| --- | --- |
| **How did you feel during the encounter?** | |
| Content aspects | Possible further enquiries |
| - Thoughts and - Feelings during the encounter | - What was going through your mind during the encounter? - How did you feel during the encounter? - What did you notice during the encounter? |

| **2.2 Climate change in encounter: setting the topic** | |
| --- | --- |
| **To what extent did climate change play a role during the encounter?** | |
| Content aspects | Possible further enquiries |
| - Climate change perception in encounter | - Was it particularly obvious/ special to you that your doctor raised climate change and mitigation issues during the encounter?   - Why do you think you (didn't) notice(d) this in particular?   - Would you have wished that your doctor had presented/shown the connection to climate more clearly?   - To what extent did you understand the connection to climate change when your doctor invited you to participate in this study? |

| **2.3 Climate change in encounter: encounter flow** | |
| --- | --- |
| **How exactly has climate change been addressed?** | |
| Content aspects | Possible further enquiries |
| - Procedure of the doctor - Transition to climate change - Directness | - How did your doctor transition to the topic?   - Were you asked if you would like to talk about the topic? - Have you received a flyer or other information material? - How long was the encounter in total and how long was climate change etc. discussed? - Outside of the encounter with your doctor, did you get the impression that the topic of climate change is present in the practice? (e.g. via posters, the website, flyers on display, via the practice staff?) |

| **2.4 Climate change in encounter: Effect during the encounter** | |
| --- | --- |
| **What was it like for you to talk about climate change during the encounter?** | |
| Content aspects | Possible further enquiries |
| - Effect of the doctor's action on   - Feelings   - Thoughts of the patient - Encounter methodology | - How did you feel when climate change was discussed? - What did the encounter about climate change trigger in you? - How did you feel about your doctor's approach? - How did you feel about your mode of communication? What did you feel was good? What did you find rather inappropriate? |

| **2.5 Climate change in encounter: meeting the patient's needs** | |
| --- | --- |
| **To what extent did you feel you were well advised in the encounter?** | |
| Content aspects | Possible further enquiries |
| - Personal interest - Relevance of the topic for the patient - Orientation towards health concerns vs. general statements - Needs of the patient | - Did you feel that your personal health concern was well discussed?   - Why did you feel well or not so well advised? - What else would you have liked from your doctor?   - Information material?   - Further talks in progress? |

| **3. Effects in the aftermath of the encounter** |
| --- |
| **When you look back now, what effect did the encounter have on you afterwards?** |
| Possible further enquiries |
| - With what feeling did you leave the encounter? - Did you think back to the encounter later? *If yes,* which aspects did you think about? - Compared to before: To what extent are you more interested in dealing with climate change after the encounter? - To what extent did the encounter create a more personal connection to climate change or climate protection? - *When worries about climate change are expressed:* How have your concerns about climate change developed due to the encounter? - To what extent did the encounter influence your political views on climate change or protection? - What happened in the days or weeks after the encounter? - How did the encounter affect your behaviour, if at all? - To what extent do you feel strengthened by the encounter to change your behaviour?   - How are the health benefits relevant to you?   - How are the positive effects on the climate-relevant for you? - What prevents you from implementing what you have discussed with your doctor? What could help you? - How did the encounter affect how you feel about your doctor? - How did the encounter affect your relationship with your doctor? |

| **4.1 Probing: Topics** |
| --- |
| **When you think about climate change, so you sometimes think: ‘This has to do with me’ or ‘This affects me personally’?** |
| Possible further enquiries |
| - What connections do you know between climate change and health? - What connections do you know between climate change mitigation and health? - *Depending on the response (heat waves, diet, exercise, etc.):*    - Which of these connections are particularly interesting for you?   - What would be helpful for you in this topic?   - Which of these topics would you like to discuss with your doctor? |

| **4.2 Probing: Appropriateness of climate change in encounter, goals of climate encounter** |
| --- |
| **Why do you think your doctor integrates the topic of climate change into the patient enounter?** |
| Possible further enquiries |
| - *Depending on the answer:* What do you think of this? / How do you feel about it? - To what extent do you feel it is appropriate for your doctor to talk to you about climate change? - To what extent do you think it is your doctor's job to educate you about these connections? - What points of conflict do you see in such a encounter? - Was politics a topic in the encounter?   - To what extent do you think the issue of climate change and health is a political issue?   - Some patients might say that climate change is too political for the doctor-patient encounter. How do you see that? - Some patients might say: ‘When I go to the doctor, I want to talk about my health, not climate change.’ How do you see that? - *Scenario 1*: How would you feel / how do you feel about your doctor wanting you to lead a more climate-friendly life by talking about climate change? (E.g., eating less meat or animal products, cycling more often instead of driving). - Some people say that by recommending eating less meat or driving less, the responsibility is transferred from politicians or companies to the individual.   - Did you get this feeling from talking to your doctor?   - What is your position on this discussion? - *Scenario 2*: Maybe your doctor lets you know that she personally supports political climate protection measures (e.g., a car-free city centre, building photovoltaic systems on roofs, building wind turbines in the countryside). How would you feel about that? - *Scenario 3*: What would you think if your doctor said: ‘Climate protection and thus the protection of our health can only really be effectively enforced at the political level. If climate change continues to progress, at some point, I will be powerless as a doctor and will no longer be able to protect my patients from the effects of climate change adequately. I think we need to change this socially and it needs all of us. How about getting involved in an initiative for more climate protection at the political level, for example, or going on a demonstration for more climate protection?’ - What do you think: Why did your doctor assess you in such a way that she can discuss the issue of climate change with you? |

| **4.3 Probing: credibility, role of the doctor as climate change communicator** |
| --- |
| **What does it mean if your doctor talks to you about climate change?** |
| Possible further enquiries |
| - To what extent do you find your doctor competent to talk about climate change? - Did your doctor seem convincing to you when she talked about climate change? Why? - If you compare the encounter about climate change with your doctor to other conversations about climate change: Are there similarities? -> What are the differences? - When you think of the doctors' group in general: Are these generally people you would listen to if they spoke out for more climate change mitigation? *If unclear, narrow down the target group:* If doctors were to speak out publicly in favour of climate protection? If doctors would speak out in favour of climate protection in encounters with patients? - How would you describe your doctor? - How would you describe your relationship with your doctor?   - To what extent has your relationship with your doctor been a factor in how the encounter about climate change has affected you? - *Filter question:* Did you know your doctor is involved or engaged in climate change and health?   - Did this knowledge of your doctor's commitment lead you to become or remain a patient with the doctor?   - *When the doctor mentions climate-friendly behaviour / commitment:* What effect does this have on you? Does it strengthen your own motivation to do X more as well? - Do you have contact with other patients of your doctor and know what they say about your doctor? |

| **Final questions** |
| --- |
| - Is there anything important to you that we haven't discussed? - *Describe the overall impression and ask for patient feedback.* - Was this interview today difficult for you in any way? *(To be able to provide support, if needed)*   **Thank you very much for the interview!** |
