# Supplementary material for: Patients’ perceptions of climate-sensitive health counselling in primary care: Qualitative results from Germany
Source: Eur J Gen Pract. 2023 Nov 27;29(1):2284261. doi: 10.1080/13814788.2023.2284261 (PMC10773651; doi:10.1080/13814788.2023.2284261)
Supplement: Supplemental Material [file IGEN_A_2284261_SM2806.docx]

**Supplementary Material 3. Recruitment process**

The physicians supporting the recruitment process identified eligible patients who had received CSHC and gave them a hand-out with information about the study and contact information of the study team. If patients agreed, they could sign a form, which allowed the physicians to transfer the contact details of the patients (phone number and/ or e-mail) to the study team. The patients who did not sign the form needed to contact the study team themselves, and the patients who provided their contact details received a phone call from a research team member (SG).

One hundred twelve patients were identified as eligible by the recruiting physicians and received a hand-out with the study and contact information. Thereof, 36 agreed and signed a form, allowing the physician to transfer the patient’s name and contact details to the study team. 32 of 36 were contacted, whereas four were not contacted because saturation had already been reached and we had captured various patients concerning their age, climate change related attitude and two out of 32 participants could not be reached. Six other participants refused to participate: three due to low time capacities, one due to illness and fear of strangers and two without further explanation. From the 112 identified patients, 76 received the hand-out but did not sign the form. Five of these patients took the initiative and contacted the study team. Due to data protection reasons, we could not get personal information about the other 71 participants, who did not contact us. Figure 1 summarises this process.


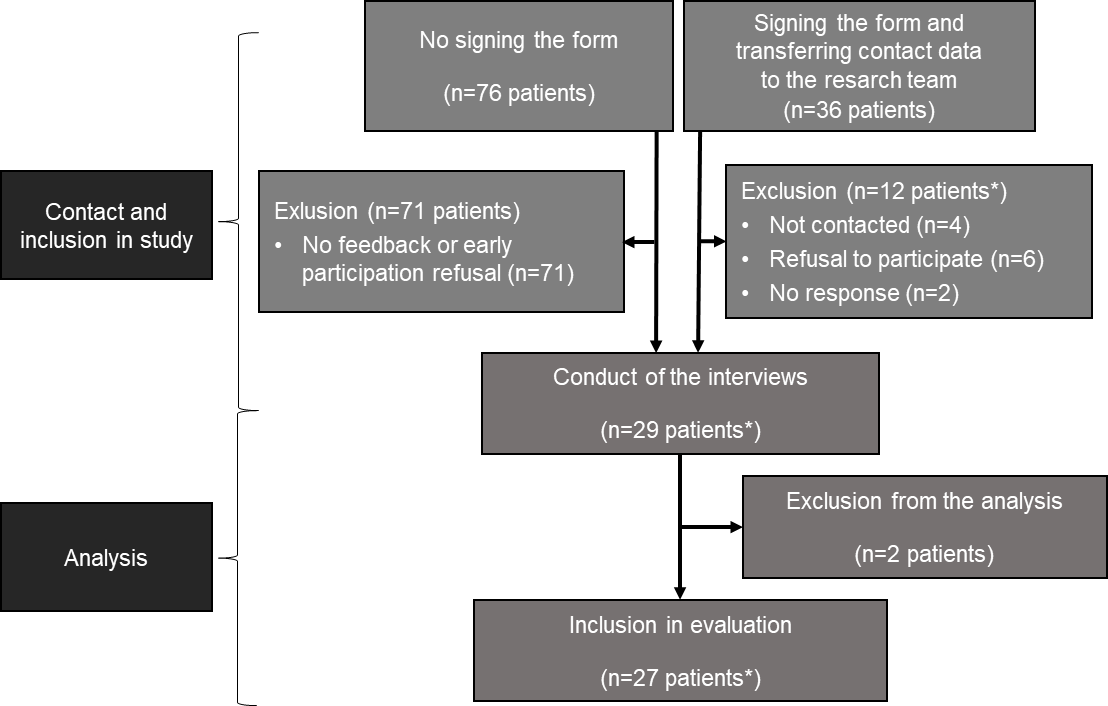


Figure 1 Final patient population. The first section of the diagram shows the recruitment phase, from contacting patients to conducting the interviews. The second section shows the generation of the final patient collective included in the analysis.

The recruitment process led to a sample of 29 patients, who had been interviewed (24/36 patients, who the study team could contact and 5/76 patients who contacted the study team themselves). As two patients could not recall discussing the climate change and health issues, they had been excluded from the analysis, leaving a final sample of 27.
