# Supplementary material for: Patients’ perceptions of climate-sensitive health counselling in primary care: Qualitative results from Germany
Source: Eur J Gen Pract. 2023 Nov 27;29(1):2284261. doi: 10.1080/13814788.2023.2284261 (PMC10773651; doi:10.1080/13814788.2023.2284261)
Supplement: Supplemental Material [file IGEN_A_2284261_SM2804.docx]

**Supplementary Material 2. Characteristics of recruiting physicians**

Table 1. Characteristics of recruiting physicians. The population density is given according to the definition of Eurostat [1] as of 2021 [2].

| **Characteristics** | **median (minimum – maximum) / percentage (*n*)** |
| --- | --- |
| Further specialist training |  |
| Specialists in general medicine | 83% (5) |
| Specialist in endocrinology and diabetology | 17% (1) |
| Male sex | 33% (2) |
| Age (years) | 56 (39 – 64) |
| Work experience (years) | 29 (14 – 40) |
| Practice location |  |
| Densly populated | 50% (3) |
| Medium populated | 33% (2) |
| Sparsley populated | 17% (1) |

1. Destatis. Definition der Stadt-Landgliederung von EUROSTAT gültig ab 31.12.20112012 28.07.2022. Available from: <https://www.destatis.de/DE/Themen/Laender-Regionen/Regionales/Gemeindeverzeichnis/Administrativ/definition-stl-ab-31122011.html>.

2. Eurostat. Lokale Verwaltungseinheiten (LAU). 2021, Available from: <https://ec.europa.eu/eurostat/de/web/nuts/local-administrative-units> [Accessed: 28.07.2022].
