# Supplementary material for: Patients’ perceptions of climate-sensitive health counselling in primary care: Qualitative results from Germany
Source: Eur J Gen Pract. 2023 Nov 27;29(1):2284261. doi: 10.1080/13814788.2023.2284261 (PMC10773651; doi:10.1080/13814788.2023.2284261)
Supplement: Supplemental Material [file IGEN_A_2284261_SM2788.docx]

**Supplementary Material 1. Pre-Interview Questionnaire**

Administered initially online and in German

Climate and health in the physician-patient consultation

Short questionnaire before the interview

Dear Participant,

The Heidelberg Institute for Global Health is conducting a study on climate change and health in doctor-patient consultation. You have chosen to participate in an interview with us as part of this study. We would like you to complete the following questionnaire to prepare for this interview. It consists of 21 questions and will take about 10 minutes to complete. This questionnaire asks for your characteristics (e.g., age, gender, education, etc.) and your views on climate change. Your information will influence the interview technique and the later analysis of the interview. Furthermore, this information serves us to get an impression of how diverse the participants in our study were during the evaluation of the interviews. This influences the generalisability and significance of our results.

Therefore, please answer as openly and honestly as possible. Some questions, which ask for sensitive information, such as income, are not obligatory and do not have to be answered if you do not wish to do so.

Your information will be treated in strict confidence and is subject to the privacy policy we have given you with the information letter and the consent form for study participation.

Note on the choice of words: In this study, we use the term ‘global warming’ synonymously with ‘climate change.’ The term ‘global warming’ or ‘climate change’ refers to the idea that the average global temperature has been rising for about 150 years may continue to grow in the future and ultimately the global climate is changing.

Thank you in advance for your participation!

I confirm that I am willing to participate in this study by proceeding to the next page.

*Data protection*

Below, you will find an excerpt from the data protection regulation, which sent to you beforehand. There you will find more detailed information. With proceeding, you confirm that:

‘I am aware that personal data will be processed during this study. The processing of the data is carried out per legal provisions and requires the following declaration of consent under Article 6(1) of the General Data Protection Regulation:

I have been informed that personal information is collected in this survey and I voluntarily consent to my information being collected and evaluated in pseudonymsed form.

‘Pseudonymised’ means that not my name/ e-mail address, but a letter code (without initials) is used for storage. The data assignment to a study participant is only possible if the key with which the data was pseudonymised is used for this purpose.

My data may be passed on to direct project staff in pseudonymised form for study purposes only. Third parties will not be given access to personal information. My name or e-mail address will not be mentioned in the publication of the study results.

I am aware that this consent can be revoked at any time in writing or verbally without giving reasons and without any disadvantages for me. This does not affect the lawfulness of the data processing carried out until the revocation. In doing so, I can decide whether the data collected from me should be deleted or may continue to be used for the study. Ultimately anonymised data cannot be traced back and thus cannot be deleted subsequently.’

If you have any questions, please contact:

*XXX (anonymised for publication)*

E-Mail: XXX *(anonymised for publication)*

Phone Number: XXX *(anonymized for publication)*

Head of study: *XXX (anonymized for publication)*

E-Mail: *XXX (anonymized for publication)*

Address: *XXX (anonymized for publication)*

Questionnaire

1. Personal Details

In this section, we ask you to provide a few details about yourself, including, for example, age, gender, school education, income, etc.

Mandatory information is marked with an asterisk (*) before the question. However, sensitive information on your income is not obligatory and does not have to be answered by you. In this case, please select ‘No answer.’

|  | 1. To which of the following age categories do you belong? * |
| --- | --- |
|  | 18-19 |
|  | 20-29 |
|  | 30-39 |
|  | 40-49 |
|  | 50-59 |
|  | 60-69 |
|  | 70-79 |
|  | 80-89 |
|  | 90-99 |
|  | 100 or older |

|  | 2. Please indicate your gender* |
| --- | --- |
|  | Female |
|  | Male |
|  | Divers |

|  | 3. What is your marital status?* |
| --- | --- |
|  | Married |
|  | Widowed |
|  | Divorced |
|  | Separate |
|  | Single |
|  | Other |

|  | 4. What is your highest educational qualification? (If you have completed another school or vocational qualification, enter this in the ‘Other’ field)* |
| --- | --- |
|  | No formal qualification |
|  | Secondary school leaving certificate, no completed vocational training |
|  | General higher education entrance qualification (Abitur), entrance qualification for studies at universities of applied sciences, subject-linked higher education entrance qualification and/or completed vocational training |
|  | University degree at bachelor level, completed master school/administrative college or similar. |
|  | University degree at Master's level (e.g. Diplom, Magister, Staatsexamen), doctorate, habilitation |
|  | Other |

|  | 5. What was the total gross income of all members of your household last year? * |
| --- | --- |
|  | 0-9.999 € |
|  | 10.000-19.999 € |
|  | 20.000-29.999 € |
|  | 30.000-39.999 € |
|  | 40.000-49.999 € |
|  | 50.000-59.999 € |
|  | 60.000-69.999 € |
|  | 70.000-79.999 € |
|  | 80.000-89.999 € |
|  | 90.000-99.999 € |
|  | 100,000 € or more |
|  | No answer |

1. What are your views on global warming or climate change

This section asks for your personal views on global warming or climate change.

Recently, you may have noticed that global warming has been getting some attention in the news. Global warming refers to the idea that the world’s average temperature has been increasing over the past 150 years may be increasing more in the future and that the world’s climate may change.

Note on the choice of words: Within this study, we use ‘climate change’ and ‘global warming’ as synonymous terms.

|  | 1. What do you think? Do you believe that global warming is happening? |
| --- | --- |
|  | Yes…and I'm extremely sure |
|  | Yes…and I'm very sure |
|  | Yes…and I'm somewhat sure |
|  | Yes…but I'm not at all sure |
|  | No…and I'm extremely sure |
|  | No...and I'm very sure |
|  | No...and I'm somewhat sure |
|  | No...but I'm not at all sure |
|  | I do not know |

|  | 2. Assuming global warming is happening, do you think it is ... |
| --- | --- |
|  | Caused mainly by human activities |
|  | Caused mainly by natural changes in the environment |
|  | Other |
|  | None of the above because global warming isn't happening |

|  | 3. How worried are you about global warming? |
| --- | --- |
|  | Very worried |
|  | Somewhat worried |
|  | Not very worried |
|  | Not at all worried |

|  | 4. How much do you think global warming will harm you personally? |
| --- | --- |
|  | Not at all |
|  | Only a little |
|  | A great deal |
|  | Don't know |

|  | 5. When do you think global warming will start to harm people in Germany? |
| --- | --- |
|  | They are being harmed now |
|  | In 10 years |
|  | In 25 years |
|  | In 50 years |
|  | In 100 years |
|  | Never |

|  | 6. How much do you think global warming will harm future generations? |
| --- | --- |
|  | Not at all |
|  | Only a little |
|  | A moderate amount |
|  | A great deal |
|  | Don't know |

|  | 7. How much had you thought about global warming before today? |
| --- | --- |
|  | A lot |
|  | Some |
|  | A little |
|  | Not at all |

|  | 8. How important is the issue of global warming to you personally? |
| --- | --- |
|  | Not at all important |
|  | Not too important |
|  | Somewhat important |
|  | Very important |
|  | Extremely important |

|  | 9. How much do you agree or disagree with the following statement: ‘I could easily change my mind about global warming.’ |
| --- | --- |
|  | Strongly agree |
|  | Somewhat agree |
|  | Somewhat disagree |
|  | Strongly disagree |

|  | 10. How many of your friends share your views on global warming? |
| --- | --- |
|  | None |
|  | A few |
|  | Some |
|  | Most |
|  | All |

|  | 11. Which of the following statements comes closest to your view? |
| --- | --- |
|  | Global warming is not happening. |
|  | Humans cannot reduce global warming, even if it is happening. |
|  | Humans could reduce global warming but people aren't willing to change their behavior, so we're not going to. |
|  | Humans could reduce global warming but it's unclear whether we will do what's needed. |
|  | Humans can reduce global warming and we will do so successfully. |

|  | 12. Do you think citizens should be doing more or less to address global warming? |
| --- | --- |
|  | Much less |
|  | Less |
|  | Currently doing the right amount |
|  | More |
|  | Much more |

|  | 13. Over the past 12 months, how many times have you punished companies that are opposing steps to reduce global warming by NOT buying their products? |
| --- | --- |
|  | Never |
|  | Once |
|  | A few times (2-3) |
|  | Several times (4-5) |
|  | Many times (6+) |
|  | Don't know |

|  | 14. Should global warming be a low, medium, high, or very high priority for the President and Congress? |
| --- | --- |
|  | Low |
|  | Medium |
|  | High |
|  | Very high |

|  | 15. People disagree about whether Germany should reduce greenhouse gas emissions on its own or make reductions only if other countries do too. Which of the following statements comes closest to your point of view? |
| --- | --- |
|  | The United States should reduce its greenhouse gas emissions ... |
|  | Regardless of what other countries do |
|  | Only if other industrialised countries (such as England, Germany and Japan) reduce their emissions |
|  | Only if other industrialised countries and developing countries (such as China, India and Brazil) reduce their emissions |
|  | The US should not reduce its emissions |
|  | Do not know |

1. Raising the issue of climate change/global warming in the physician-patient consultation

In our study, we would like to interview patients who have talked to their physicians directly or indirectly about the links between climate change and health.

The word ‘climate change’ may not have been mentioned or the conversation may have been brief. It may, therefore, be difficult to determine whether you have talked about climate change with your physician.

Note: If you tick ‘No,’ this will not affect whether we want to interview you. Therefore, answer to the best of your knowledge and belief.

|  | 1. Have you ever talked to your physician about global warming/climate change?* |
| --- | --- |
|  | YES, once |
|  | YES, a few times (2-3) |
|  | YES, several times (4-5) |
|  | YES, many times (6+) |
|  | No |
|  | I do not know |

Dear Participant,

Your data has been saved and transmitted.

Thank you for filling in the questionnaire!

I look forward to the upcoming conversation or interview with you.

With kind regards

*XXX (anonymised for publication)*

For questions and feedback you can contact me at:

*XXX (anonymised for publication)*
